# Mineralogy of microbially induced calcium carbonate precipitates formed using single cell drop-based microfluidics

**DOI:** 10.1038/s41598-020-73870-y

**Published:** 2020-10-16

**Authors:** Neerja M. Zambare, Nada Y. Naser, Robin Gerlach, Connie B. Chang

**Affiliations:** 1 Department of Chemical and Biological Engineering, Montana State University, Bozeman, MT 59717 USA; 2 Center for Biofilm Engineering, Montana State University, Bozeman, MT 59717 USA; 3 Department of Chemical Engineering, University of Washington, Seattle, WA 98195 USA

**Keywords:** Fluorescence imaging, Environmental biotechnology, Biogeochemistry, Confocal microscopy, Scanning electron microscopy, Transmission electron microscopy

## Abstract

Microbe-mineral interactions are ubiquitous and can facilitate major biogeochemical reactions that drive dynamic Earth processes such as rock formation. One example is microbially induced calcium carbonate precipitation (MICP) in which microbial activity leads to the formation of calcium carbonate precipitates. A majority of MICP studies have been conducted at the mesoscale but fundamental questions persist regarding the mechanisms of cell encapsulation and mineral polymorphism. Here, we are the first to investigate and characterize precipitates on the microscale formed by MICP starting from single ureolytic *E. coli* MJK2 cells in 25 µm diameter drops. Mineral precipitation was observed over time and cells surrounded by calcium carbonate precipitates were observed under hydrated conditions. Using Raman microspectroscopy, amorphous calcium carbonate (ACC) was observed first in the drops, followed by vaterite formation. ACC and vaterite remained stable for up to 4 days, possibly due to the presence of organics. The vaterite precipitates exhibited a dense interior structure with a grainy exterior when examined using electron microscopy. Autofluorescence of these precipitates was observed possibly indicating the development of a calcite phase. The developed approach provides an avenue for future investigations surrounding fundamental processes such as precipitate nucleation on bacteria, microbe-mineral interactions, and polymorph transitions.

## Introduction

Interactions between microbes and minerals are a central aspect of geomicrobiology, with many of these interactions having reciprocal consequences. Mineral formation and dissolution can affect microbial processes such as nutrient acquisition^1^, energy generation^1^, and biofilm structure^2^; microbial processes can, in turn, affect mineral formation, transformation, and dissolution^1^. Numerous geomicrobiological processes in nature involve interactions between bacteria and minerals, such as heavy metal mobility^3,4^, pollutant bioavailability^3,4^ and sediment magnetization^5^. Microbe-mineral interactions (MMI) play a fundamental role in rock formation and weathering^1^, acid mine drainage^6^, and hydrothermal vent formations^7^. While geomicrobiological processes can be readily observed at the macroscale, they are driven by cellular and enzymatic reactions that occur at the micro- or even nanoscale^1^. Studying microbe-mineral interactions at the single-cell level (micro- to nanoscale) can provide insight into the underlying mechanisms behind large-scale processes^8^.

One example of MMI is microbially induced calcium carbonate precipitation (MICP), a biomineralization process that occurs both in nature and in engineered systems as a result of bacterial activity^9^. MICP can occur when a metabolic process (such as sulfate or nitrate reduction, urea hydrolysis, or photosynthesis) results in an increase of pH and alkalinity, raising the saturation state of calcium carbonate (CaCO_3_) and causing CaCO_3_ precipitation^9 ^. Equation (1) summarizes the hydrolysis of urea by the enzyme urease. The produced ammonia, at circumneutral pH, functions as a Brønsted-Lowry base forming ammonium and hydroxyl ions, increasing the pH (Eq. 2). This increase in pH results in an increase in bicarbonate ions (Eq. 3), increasing saturation and promoting calcium carbonate precipitation (Eq. 4). Equation 5 summarizes the overall reaction, resulting in two moles of ammonium generated and one mole of calcium carbonate precipitated per mole of urea hydrolyzed.

1 $$CO\left( {NH_{2} } \right)_{2} + 2H_{2} O {\underrightarrow {urease}}\ 2NH_{3} + H_{2} CO_{3}$$

2 $$2NH_{3} + 2 H_{2} O \to 2NH_{4}^{ + } + 2OH^{ - }$$

3 $$H_{2} CO_{3} + OH^{ - } \to HCO_{3}^{ - } + H_{2} O$$

4 $$Ca^{2 + } + HCO_{3}^{ - } \to CaCO_{3\left( s \right)} + H^{ + }$$

5 $$CO\left( {NH_{2} } \right)_{2} + 2H_{2} O + Ca^{2 + } \underrightarrow {urease}\ 2NH_{4}^{ + } + CaCO_{3\left( s \right)}$$

Visualization of MMI is typically performed on samples extracted from bench-scale reactor systems, which may not be accurate representations of the original state of the microbes or minerals. Such ‘ex situ’ visualizations do not allow for non-destructive, time-resolved observation of microbes and minerals starting from single cells or small groups of cells. Non-invasive visualization of MICP at the nano- and microscale has the potential to provide temporally resolved, quantitative comparisons of mineral nucleation, mineral growth, and mineral types. These comparisons are important in understanding MMI and in linking the biogeochemical reactions occurring at the nano- and microscale to the macroscale. One microbe-mineral interaction believed to occur during MICP is the encapsulation of ureolytic cells by the CaCO_3_ precipitates produced^10–13^, and a decrease in ureolytic activity after precipitate formation, observed in bulk MICP experiments, is often attributed to cell encapsulation^10,14^. While bulk chemistry changes can be good indicators of cell activity, a direct correlation to encapsulation might not be accurate. Recently, Zhang et al. argued that nucleation at the cell surface might be an oversimplified model and called for direct observations of the nucleation process^15^. Cell encapsulation during biological precipitation has been suggested by several authors^10–13^ but has primarily been inferred from electron micrographs of ex situ samples. Electron micrographs generally show precipitates with indentations that match the dimensions of ureolytic cells, or hollow tubular structures approximately the diameter of bacterial cells leading to the hypothesis of the cells becoming entombed in the precipitates. However, sample preparation for electron microscopy often results in considerable changes of the physical structure of the sample. Dehydration during sample preparation and electron microscopy can cause the sample to shrink, distort and can give rise to other artifacts that might not be representative of the original state^16^. Thus, in situ visualization of MICP at the microscale in real-time is required to investigate the potential for cell encapsulation as well as other MMIs.

Non-invasive visualization of MMIs during biological calcium precipitation at the single-cell level can be achieved by drop-based microfluidics. Single cells can be isolated in picoliter-sized drops that are tens of micrometers in diameter; these drops are formed at rates of thousands per second by flowing fluids of an aqueous phase and an oil phase into a flow-focusing microfluidic device that creates drops of water in oil^17^. When bacterial cells are isolated within drops^18^, the drops serve as microscopic bioreactors, allowing studies on microbial growth, kinetics, fundamental cellular physiology, and enzyme kinetics^19–21^. Drop-based microfluidics has been used to observe abiotic precipitation processes^22–24^ but to our knowledge has never been used to study MICP starting from single bacterial cells. Drop-based microfluidics can enable high-resolution and time-resolved observation of biomineralization at the nano- to microscale. The ability to study biomineralization in a non-invasive manner can provide valuable insights into the mechanisms underlying large scale precipitation processes and contribute to designing novel bio-based materials.

Here, we visualized ureolysis-driven MICP by single *E. coli* MJK2^25^ cells using drop-based microfluidics. We generated 25 µm diameter drops containing single MJK2 cells in media and observed the growth of cells and CaCO_3_ minerals over time. We varied the calcium ion concentration and found that calcium carbonate precipitates were observed in drops at all concentrations if urea and MJK2 cells were present. High calcium ion concentration in the media decreased cell growth and cell motility. In these experiments, precipitates were also observed in neighboring drops without ureolytic cells indicating drop-drop exchange that facilitated precipitation in drops without ureolytic cells. Precipitates that formed inside drops were characterized using a range of microscopy and microanalysis techniques. We observed two distinct polymorphs of CaCO_3_ precipitates in drops, vaterite and amorphous calcium carbonate (ACC). The ratio of drops containing vaterite to drops containing ACC changed over time and was dependent on the initial ratio of calcium ion and urea concentrations, reaching a maximum at an equimolar calcium ion to urea ratio. The precipitates identified as vaterite using Raman microspectroscopy exhibited two different morphologies (dense core and grainy rind) after focused ion beam milling scanning electron microscopy (FIB-SEM). These precipitates also exhibited autofluorescence, indicating the possible presence of calcite nanocrystals. This work investigates MICP formation starting from single cells, with a focus on differences in cell growth, cell motility and precipitate polymorphs as a function of time and calcium ion concentration available in the media. The methods presented here offer a platform for studying microbe-mineral processes starting from single bacterial cells, including biological precipitate formation, dissolution and polymorph transitions.

## Experimental methods

### Media and bacterial cell culture

*Escherichia coli* MJK2, a strain engineered to express GFP and produce urease^25^, was used to promote MICP. Growth medium for MJK2 was Luria–Bertani medium (BD Difco, Fisher Scientific, NH, USA) amended with 10 μg mL^−1^ gentamicin, 50 mM L-arabinose and 10 μM nickel chloride (Sigma-Aldrich, MO, USA)^25^. The cell culture was prepared by adding 50 μL of a MJK2 frozen stock culture to 10 mL of growth medium in a 50 mL centrifuge tube loosely covered with aluminum foil. The centrifuge tube was placed upright on a horizontal shaker operating at 150 RPM at ambient temperature (24.5 ± 1.5 °C). After 24 h, the optical density (OD) was measured using a BIOTEK Synergy HT spectrophotometer (600 nm wavelength, 200 μL samples in a 96-well plate, with the blank well OD of 0.044 as reference) and adjusted to an OD of 0.4 using growth medium. An additional dilution was necessary to generate drops with 1 in 10 drops exhibiting a single cell per drop. Through a series of cell enumeration experiments (see Supplementary Information and Supplementary Fig. S1), a 1:5 dilution was determined to be optimal for this purpose.

Urea and calcium chloride dihydrate (Fisher Scientific, NH, USA) were added to growth medium at the concentrations listed in Table 1 to prepare media for the six conditions tested in this study. Three conditions (Ca_Low_, Ca_Int_ and Ca_High_) had the prerequisites for MICP to occur (dissolved calcium, dissolved urea, and MJK2, *cf*. Equations 1–5); the other three conditions were negative control experiments (marked with ‘*’ in Table 1), either without calcium in the medium (Ca^2+^-free), without urea in the medium (urea-free) or without bacteria (bacteria-free). All media were filter-sterilized using Nalgene Rapid-Flow 0.45 µm pore size bottle-top filters (ThermoFisher Scientific, MA, USA). The pH of the media at the time of drop-generation was 6–6.3, ensuring no abiotic precipitation prior to the experiments.

**Table 1:** Experimental conditions for the drop studies.

Experiment description	Ca^2+^ (M)	Urea (M)	Calcium-to-urea molar ratio (Ca^2+^:Urea)	MJK2 cells in drops
Low Ca^2+^ (‘Ca _Low_’)	0.0825	0.165	0.5:1	Yes
Intermediate Ca^2+^ (‘Ca_Int_’)	0.165	0.165	1:1	Yes
High Ca^2+^ (‘Ca_High_’)	0.33	0.165	2:1	Yes
Ca^2+^-free*	0	0.165	–	Yes
Urea-free*	0.165	0	–	Yes
Bacteria-free*	0.165	0.165	1:1	No

The three experiments marked with ‘*’ are control experiments.

### Drop-generation and work-flow

**Figure 1:**
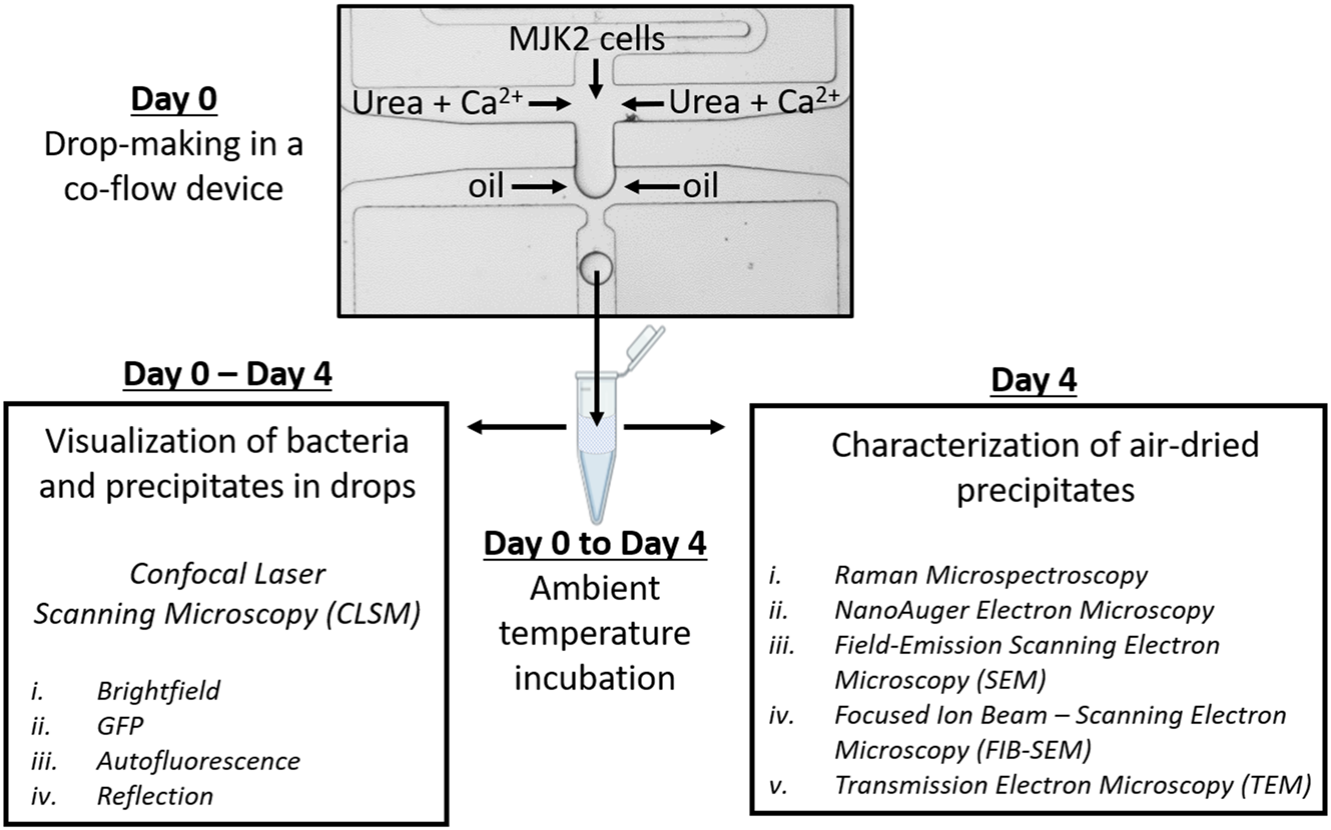
Schematic of experimental workflow showing drop generation, storage, visualization of drops and characterization of air-dried precipitates.

Co-flow microfluidic devices made of polydimethylsiloxane (PDMS) were fabricated in-house using standard soft lithography techniques^20,26^. Drops of 25 µm diameter were generated by flowing the 1:5 diluted bacterial culture, media, and Novec HFE-7500 fluorocarbon oil (3 M, St. Paul, MN, USA) with 1.5% (w/w) 008-FluorSurfactant (RAN Biotechnologies)^27^ in a flow-focusing, co-flow microfluidic device (Fig. 1). The bacterial culture plus fresh medium formed the inner phase of the water-in-oil emulsion and the oil containing surfactant formed the outer phase. Using calibrated syringe pumps, the bacterial culture and media were set to flow rates of 200 µL h^−1^ each, and the oil was set to a flow rate of 800 µL h^−1^. Drops were collected in 1.5 mL microcentrifuge tubes and stored at ambient temperature for experimentation over the next 4 days (Fig. 1).

### Bulk experiments

Bulk experiments were performed to compare cell growth in drops to cell growth in larger volumes and ureolysis at the different calcium ion concentrations. For bulk experiments, 10 mL of media were used, and the volume of bacterial inoculum added was designed to simulate the cell-to-media ratio in the drops at the time of drop generation. Bulk experiments were conducted in triplicate in 50 mL centrifuge tubes loosely covered with aluminum foil and incubated upright on a horizontal shaker (150 RPM) at ambient temperature. Fluorescence measurements (excitation 485 ± 20 nm, emission 528 ± 20 nm) were performed at sampling times consistent with drop imaging times using a BIOTEK Synergy HT spectrophotometer on 200 µL samples (reference blank well fluorescence reading of 88 fluorescence units). Samples were also diluted using 5% nitric acid (Fisher Scientific, NH, USA) for urea concentration measurement using the colorimetric Jung assay^28^. Sample pH was measured using a Symphony SB70P pH meter (VWR, PA, USA).

### Microscopy and microanalysis

#### Time-resolved analysis in drops

From the time of drop generation (Day 0) to the end of the experiment at 96 h (Day 4), drops were imaged every 24 h, for a total of 5 time points in all experiments except in the bacteria-free control, for which images were taken on days 0 and 7. In all experiments, an aliquot of drops large enough to fill a DropSOAC microfluidic device^20^ was removed from the microcentrifuge tube for imaging using an inverted confocal laser scanning microscope (CLSM, Leica SP5) at ambient temperature. During imaging, the hydrated oil-soaked DropSOAC device was submerged in hydrated oil to maximize drop stability^20^. Drops were imaged from randomly chosen locations in the DropSOAC device using a 20 × air objective and the following illumination channels:

(i) Brightfield image of the entire drop (transmission images), 168 gain, 60 µm pinhole
(ii) GFP (Excitation: 488 nm laser, Emission: 490—590 nm) to detect MJK2 cells, 612 gain, 200 µm pinhole
(iii) UV (Excitation: 405 nm laser, Emission: 415 nm—550 nm) to detect calcite through its autofluorescence^29,30^, 600 gain and 200 µm pinhole
(iv) Reflection light (488 nm incident laser, detection between 478 nm—498 nm) to detect precipitates (no excited fluorophore), 200 gain, 60 µm pinhole. A reflection signal allows visualization of precipitates as they are generated when the incident laser reflects off solid material.

The CaCO_3_ fluorescence was termed ‘autofluorescence’ as it did not require the addition of a fluorophore for signal generation. Image acquisition parameters for CLSM (gain and pinhole aperture) were kept consistent between experiments for each channel and the initial fluorescence of the 0.4 OD-adjusted cultures used for the different experiments was comparable (relative standard deviation < 2%). The single-plane images taken with a large 200 μm pinhole aperture captured signal from a greater imaging volume. This method captures signal from out-of-focus planes, allowing rapid imaging of an entire drop while preventing signal overestimation from motile cells appearing in more than one plane during Z-stack acquisition. Drops containing cells were detected using the ImageJ TrackMate plug-in^31^ by specifying 25 μm as the drop detection diameter, centered around the GFP signals on GFP channel images (visually checked against the corresponding brightfield images) and the fluorescence intensity per drop was measured. The numbers of drops analyzed per measurement are provided in Supplementary Table S1. For motility measurements, a 30 s acquisition video of approximately 300 drops from each experiment was analyzed for cell swimming speed in terms of mean track velocity (μm s^−1^) using TrackMate. Tracks were detected in drops containing cells based on GFP signal detection constrained to 5 µm diameter (numbers of tracks in Supplementary Table S2). For cell-chains (Day 1 and Day 2 for Ca_Int_) the diameter constraint was set to 10 µm. TrackMate-based drop detection was performed centered around signals from reflection channel images to count drops containing precipitates. Different diameter settings for detection were used to differentiate between drops containing the two observed precipitate morphologies (see Supplemental Information for details).

#### Precipitate characterization

On day 4, precipitates from drops were collected for visualization and determination of elemental composition and precipitate mineralogy. A 10 μL sample of drops was air-dried at ambient temperature on a glass slide overnight, breaking the drop emulsion and exposing the precipitates. The dried sample was transferred onto sticky carbon tape on a metal stub, sputter coated with iridium at 20 mA for 1 min and imaged using a Zeiss SUPRA 55VP Field Emission Scanning Electron Microscope (SEM). The same sample was analyzed using energy dispersive X-ray spectroscopy (EDX) to characterize the elemental composition of the precipitates (Scanning Auger Electron Nanoprobe, Nano-Auger, Physical Electronics 710). In addition, air-dried precipitates from each mineralization experiment were analyzed using Raman microspectroscopy (Horiba LabRAM HR Evolution NIR). Samples were also analyzed using a Focused Ion Beam-Scanning Electron Microscope (FEI Helios Nanolab dual-beam FIB-SEM) and a high-resolution Scanning Transmission Electron Microscope (aberration-corrected Titan 80–300 STEM). Samples for FIB-SEM were prepared by placing air-dried sample on a metal SEM stub and coating with carbon. For TEM, sterile water was added to air-dried drop samples and precipitate suspensions were transferred onto lacey carbon grids before being allowed to air-dry.

## Results and discussion

**Figure 2:**
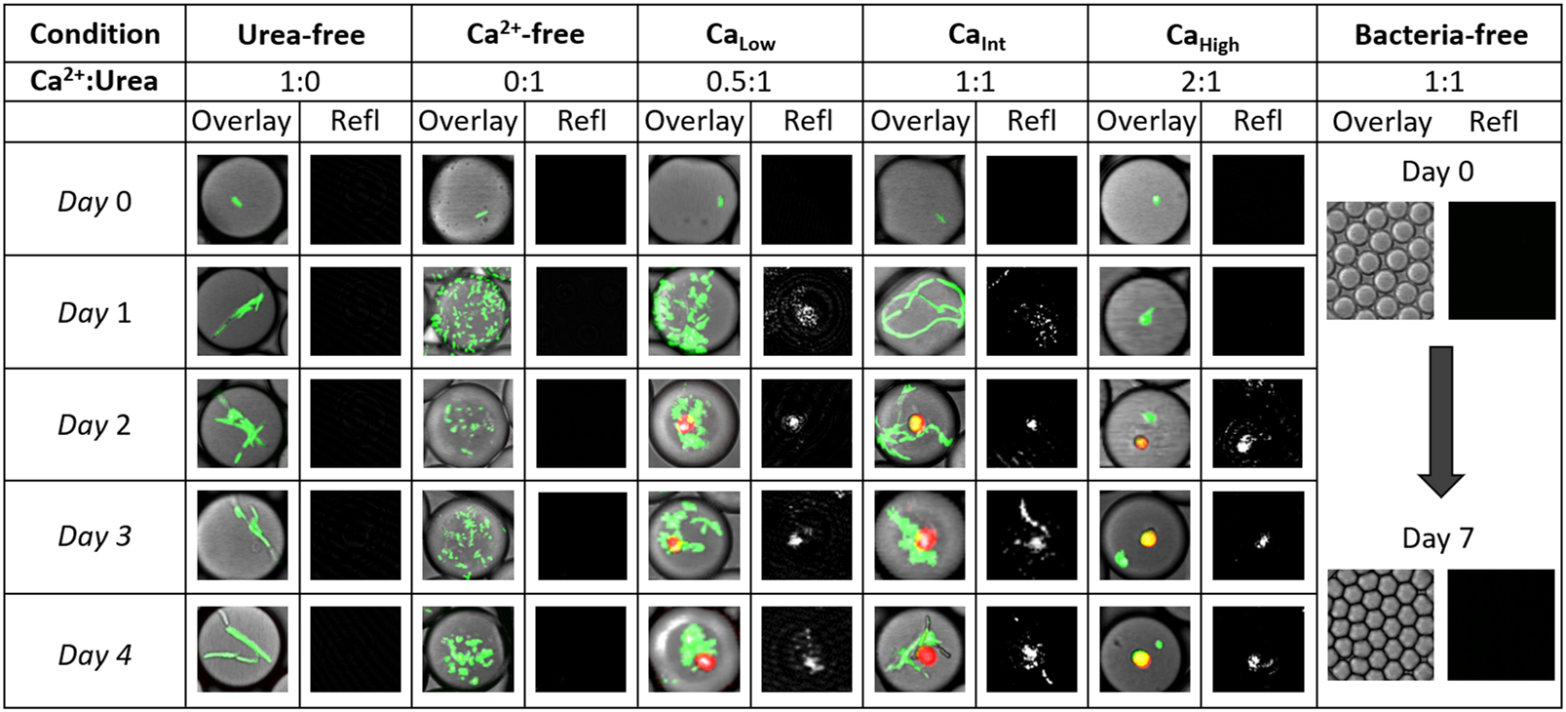
Images of drops from each experiment (conditions outlined in Table 1). The column ‘Overlay’ shows overlaid images of channels (i) brightfield, (ii) GFP and (iii) UV listed in methods while column ‘Refl’ shows the corresponding reflection signal (channel (iv) reflection outlined in “Methods” section). For the bacteria-free condition, the images are from day 0 and day 7. Individual drops shown are approximately 25 µm in diameter.

**Figure 3:**
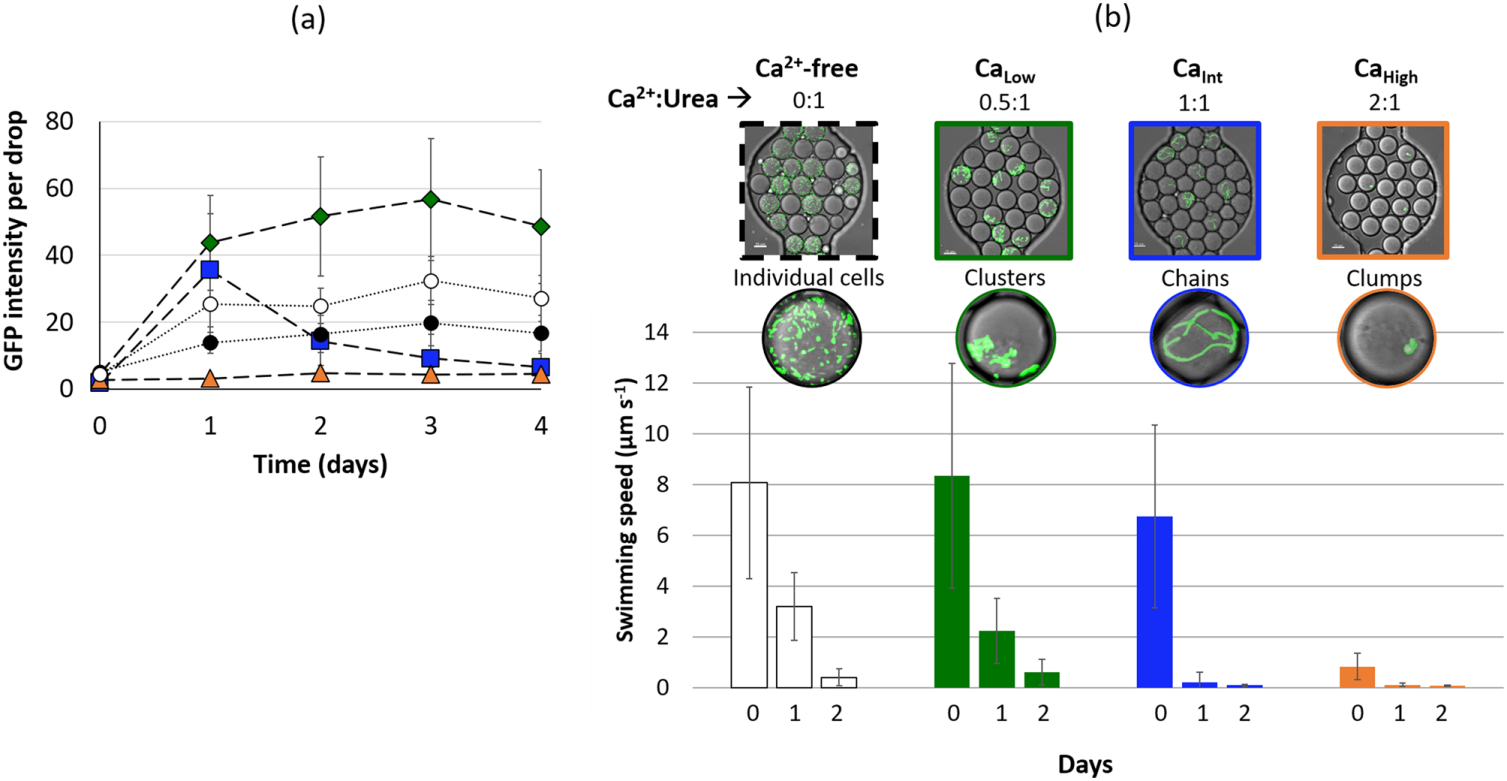
Cell growth and motility data from drop experiments. (**a**) Cell growth in drops, measured as GFP intensity per drop for Ca^2+^-free (white circles), Ca_Low_ (green diamonds), Ca_Int_ (blue square) and Ca_High_ (orange triangles); Fig. S2 provides a correlation of bacterial growth (measured as optical density) and GFP fluorescence. (**b**) Average swimming speed of cells in drops on days 0, 1, and 2 for Ca^2+^-free (white), Ca_Low_ (green), Ca_Int_ (blue) and Ca_High_ (orange) treatments. After day 2, the development of precipitates did not allow for reliable measurements of cell swimming speeds.

We observed MICP starting from single cells of *E. coli* MJK2 in 25 µm diameter drops at various concentrations of calcium ions (Fig. 2). The cells performed ureolysis, which led to an increase in alkalinity and subsequent precipitation of CaCO_3_ according to the reaction scheme outlined in Eqs. (1–5). The three control experiments, urea-free, Ca^2+^-free, and bacteria-free, did not produce precipitates and confirmed that the precipitates formed inside the drops in all mineralization experiments were the result of ureolytic MICP. The ‘Overlay’ columns in Fig. 2 display overlays of the brightfield, GFP and UV signals and the ‘Refl’ columns show the corresponding reflection signal images. The green GFP signal from MJK2 cells increases over time (*cf*. Figure 3a for quantitative analysis), and a red autofluorescence signal emanating from some of the precipitates appears beginning on day 2 in the Ca_Low_, Ca_Int_, and Ca_High_ drops.

### Cell growth, aggregation, and motility

Bacterial growth occurred in the drops as evident from an increase in the GFP intensity per drop (Figs. 2, Fig. 3a). The GFP signal over time in drops showed trends similar to the corresponding bulk GFP measurements for all conditions except Ca_Int_ (Supplementary Figs. S3 and S4). In the Ca_Int_ drops, formation of denser precipitates likely affected fluorescence measurements in drops on the inverted CLSM due to the optical configuration (see Supplemental Information), leading to the observed decrease in GFP intensity per drop in Ca_Int_ on and after day 2. Depending on the calcium ion concentration in drops, different types of cell aggregates, including clusters, chains, and clumps, were observed on day 1 (Figs. 2,  3b). In Ca_Low_ drops cells appeared as clusters, cells in Ca_Int_ drops formed long chains, while cells in Ca_High_ drops appeared to be present in tight clumps identifiable as bacteria by their GFP signal. In comparison, cells in the Ca^2+^-free drops did not aggregate, and the drops appeared full of individual cells. Cell aggregation was not further investigated, but has been previously observed for *E. coli* K12 (MJK2 is a K12 derivative)^32^.

The initial (Day 0) cell swimming speed observed in drops was variable between calcium conditions; conditions with higher calcium ion concentrations exhibited reduced average swimming speed even on day 0 (Fig. 3b). Cell motility decreased drastically between day 0 and day 1 in all experiments and continued to decrease. After day 2, cell motility could not be reliably quantified because, in many cases, cells became attached to precipitates.

### Precipitation in drops

**Figure 4:**
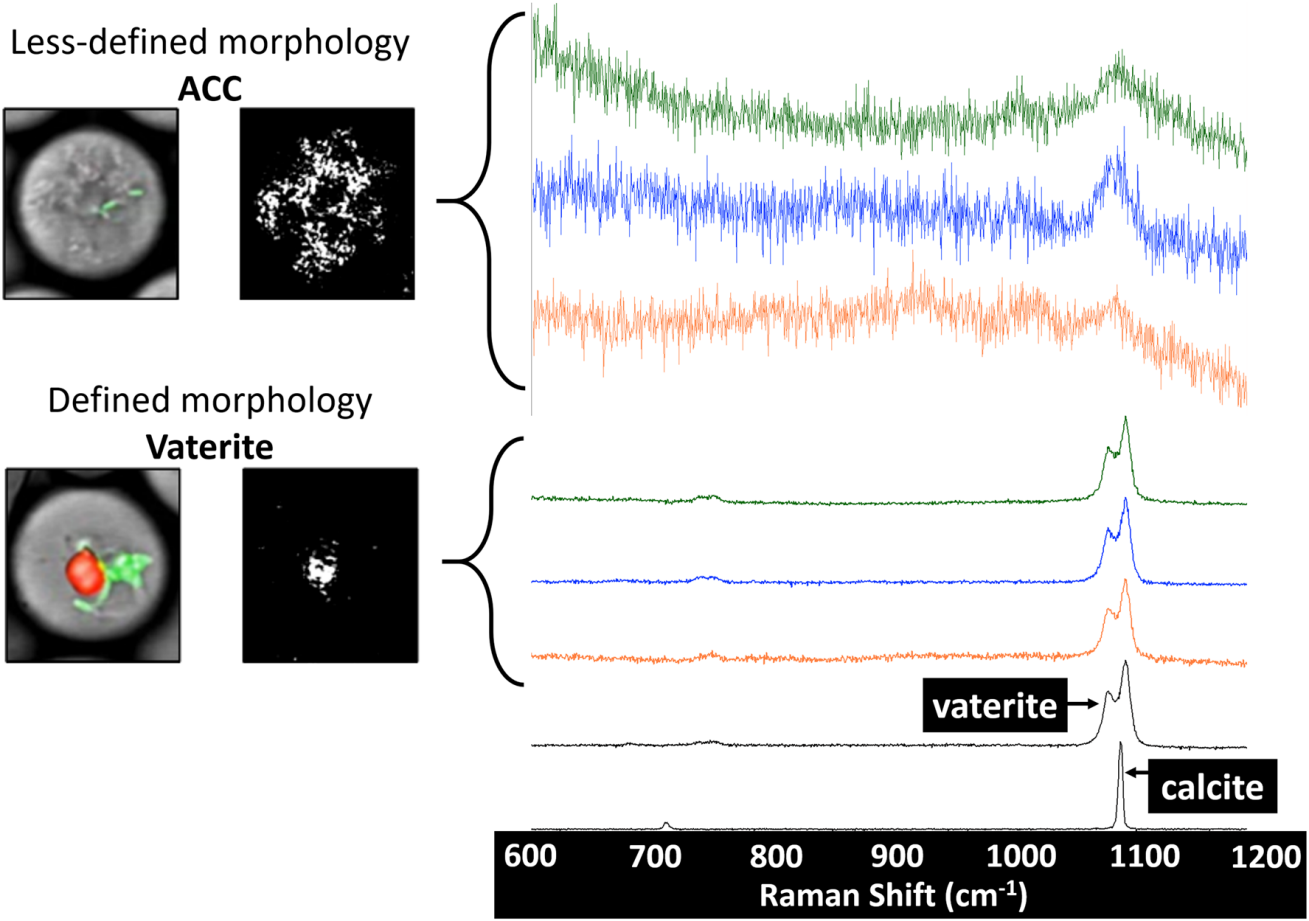
Characterization of precipitate morphologies. Raman microspectroscopy data collected from both precipitate morphologies (CLSM example images shown on the left, drops are approximately 25 µm in diameter) observed in Ca_Low_ (green), Ca_Int_ (blue) and Ca_High_ (orange). Amorphous calcium carbonate was identified by a weak Raman signal at approximately 1084 cm^-1^, consistent with published Raman spectra for ACC^24,34,35^. The denser precipitates exhibited Raman spectra with double peaks identical to those of the vaterite standard and published data^24,36,37^. An ACC standard is not shown here as it was not available and stable ACC could not be generated via experiments for use as a standard. The vaterite standard shown here was generated experimentally and was identified as vaterite using XRD and Raman microspectroscopy.

**Figure 5:**
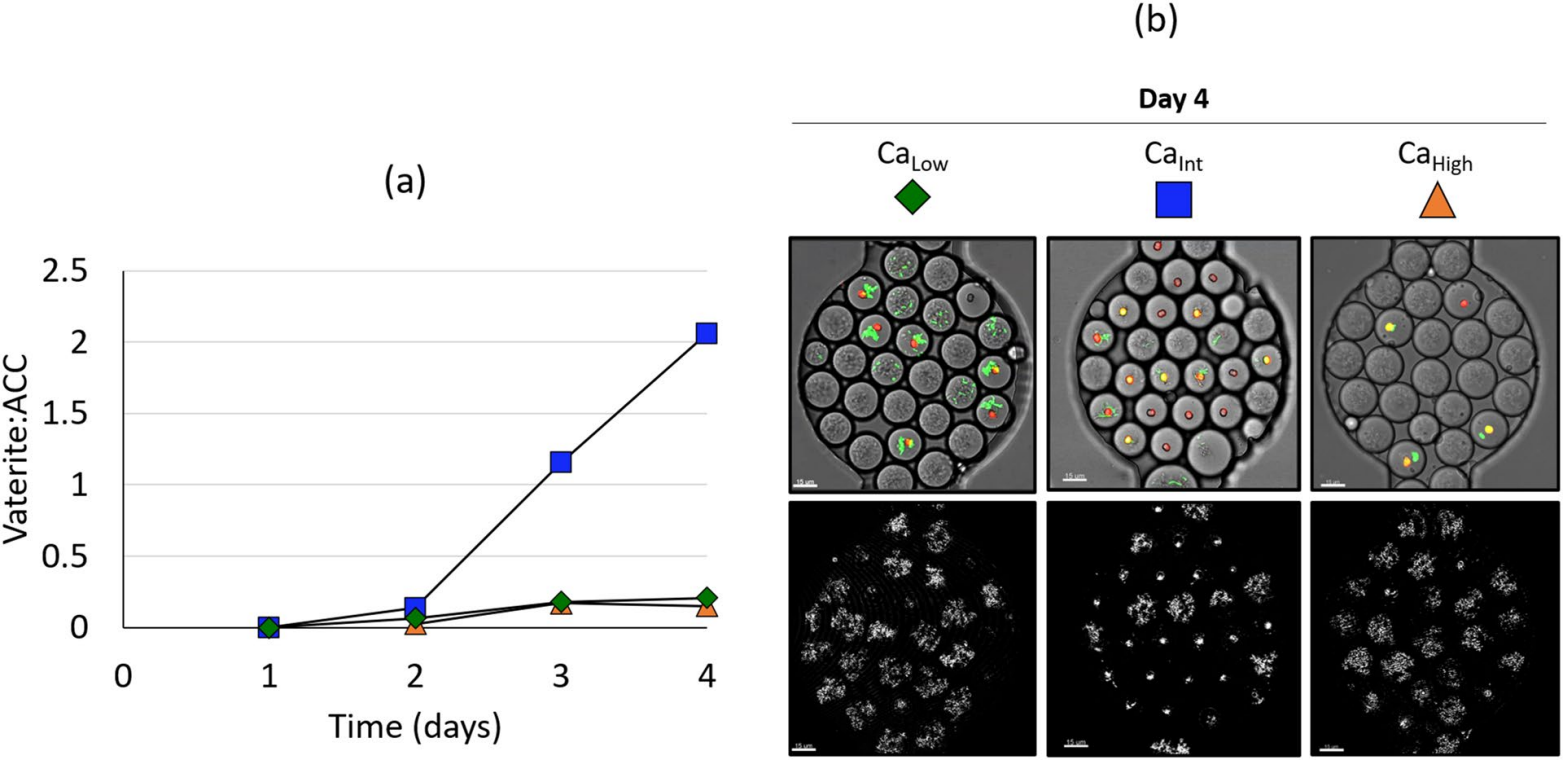
Quantification of precipitates in drops. (**a**) Vaterite:ACC ratio over time for the mineralization experiments. (**b**) Image overlays of Day 4 drops from Ca_Low_ (green diamonds), Ca_Int_ (blue squares) and Ca_High_ (orange triangles) experiments showing precipitates in drops with cells (green signal) and without cells. Scale bars are 15 µm.

Two morphologies of precipitates were observed in the drops and were distinguishable via reflection imaging (Fig. 4). The precipitates exhibiting a less-defined morphology resembled previously published images of amorphous calcium carbonate^24,33^ and Raman signals collected from these precipitates showed a broad peak at 1084 cm^−1^ (Fig. 4), which is similar to previously published Raman wavenumbers (cm^−1^) for ACC (1084^24^, 1077^34^ and 1083^35^). In contrast, the more defined precipitates revealed a Raman signal indicative of vaterite (Fig. 4) based on the characteristic double peak for vaterite at 1078 and 1092 cm^−1^, comparable to literature values of 1074, 1090 cm^−1^ ^36,37^, 1076, 1092 cm^−1^ ^24^, and to a lab-generated vaterite standard (Supplementary Fig. S8). These precipitates had an autofluorescence signal at 405 nm excitation in addition to a reflection signal indicating an additional mineral phase associated with these precipitates since to our knowledge, vaterite itself has not been observed to autofluoresce. In all mineralization experiments, ACC precipitates appeared before vaterite precipitates in the drops and no instances were observed where both ACC and vaterite were present in the same drop, suggesting that vaterite was forming at the expense of ACC (Fig. 5).

Drops containing either ACC or vaterite were quantified from reflection images in all mineralization experiments. The ratio of number of drops containing vaterite to number of drops containing ACC (Vaterite:ACC) increased over time (Fig. 5a) and this increase was most pronounced for Ca_Int_ drops. This condition contained an equimolar ratio of calcium and urea and displayed the highest number of vaterite precipitates compared to the other two Ca^2+^:Urea ratios tested (Ca_Low_ and Ca_High_), which showed comparable increases in Vaterite:ACC ratios.

Precipitates were detected later in Ca_High_ (day 2) compared to Ca_Low_ and Ca_Int_ (day 1). The delayed onset of precipitation in the Ca_High_ drops potentially resulted from lower ureolytic activity due to decreased cell growth at high calcium ion concentrations (Fig. 3a). In fact, bulk experiments indicated the slowest ureolysis rates in Ca_High_ treatments (Supplementary Fig. S6).

In drops with cells, the cells were closely associated with either the ACC or vaterite precipitates (Supplementary Video S1). Interestingly, in Ca_Low_, Ca_Int_ and Ca_High_ drops, precipitates (ACC and vaterite) were also observed in drops that exhibited no GFP signal and likely had no cells (Fig. 5.b). Control experiments demonstrated that all three MICP prerequisites (bacteria, urea and Ca^2+^) were necessary for precipitation to take place, hence precipitation in individual drops without cells suggested that these drops formed precipitates as a result of exchange of ureolytic byproducts (e.g. carbonate and/or hydroxyl ions) between drops with actively ureolyzing bacteria and drops without cells. This hypothesis was proven by an additional experiment designed to reveal exchange between drops (Supplementary Information Fig. S7). In this experiment Ca_Int_ drops (containing urea, MJK2 cells and dissolved calcium) were mixed at a 1:1 volume ratio with drops containing 1 µm diameter polystyrene fluorescent microbeads and dissolved calcium, but no urea or MJK2 cells. Precipitates were observed in drops containing bacteria as expected, but also in drops containing fluorescent microbeads without bacteria. Without exchange, the drops containing fluorescent microbeads could not have developed precipitates as they did not contain bacteria or urea, two of the three prerequisites for MICP. Drop fusion was ruled out as a major reason for the appearance of precipitates in drops without bacteria because (i) no GFP-signal, indicative of cells, was detected in these drops and (ii) the diameter of the drops analyzed was similar, on average 6% smaller, on day 4 compared to day 0. Determining the exact mechanism of transport between drops is beyond the scope of this paper but two possible mechanisms include micellar transport via reverse micelles^38,39^ and concentration gradient-based diffusion across drop-drop interfaces^40^.

**Figure 6:**
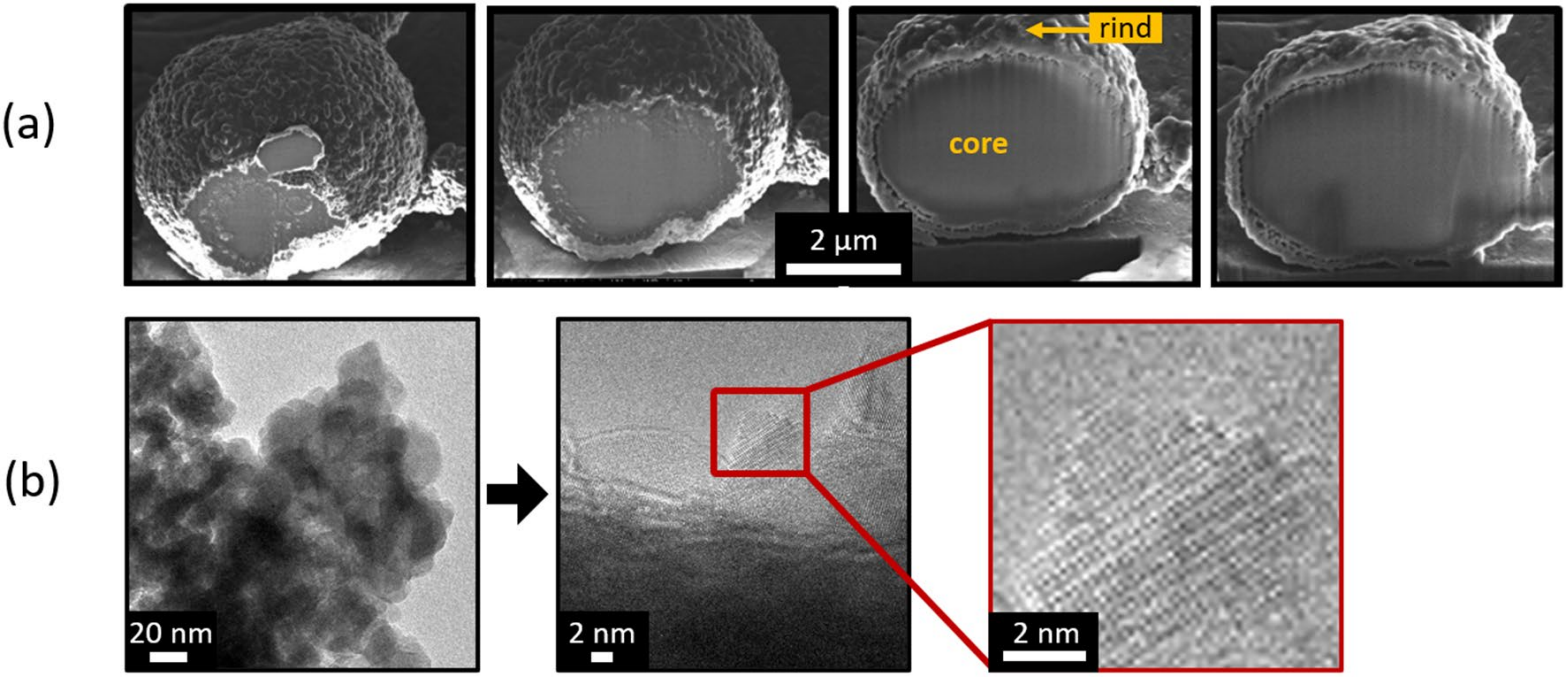
Visualization of a single vaterite precipitate using FIB-SEM and TEM. (**a**) Left to Right: SEM images showing progression of FIB milling of a single precipitate from a Ca_Int_ drop. The interior core of the precipitate appears densely packed, surrounded by a grainy rind. (**b**) TEM images of a precipitate from a Ca_Int_ drop showing atomic column structures suggestive of crystallinity.

Fluorescence emission scans (405 nm excitation, emission range: 400 nm to 600 nm) performed on 6 randomly chosen vaterite precipitates indicated a peak emission wavelength (for autofluorescence) of 481.5 nm ± 6.6 nm (Supplementary Fig. S9). Based on published data for calcite fluorescence^30^, the measured peak emission wavelength of 481.5 nm coincides with the maximum fluorescence region described for calcite. However, Raman microspectroscopy and XRD-based characterization of these precipitates indicated vaterite as the major mineral phase. Hence, one precipitate isolated from a Ca_Int_ drop was milled layer-by-layer using a Focused Ion Beam and imaged simultaneously with a Scanning Electron Microscope (FIB-SEM). The FIB-SEM progression images in Fig. 6a show that the precipitate consisted of a core covered with a grainy rind (Fig. 6a). Transmission Electron Microscopy (TEM) performed on the rind of another precipitate from a Ca_Int_ drop suggested that the rind was crystalline (Fig. 6b) but diffraction patterns did not match any known CaCO_3_ polymorphs. Beam damage during TEM resulting in calcium carbonate transformation is fairly common^41,42^ and could be the reason for the inconclusive TEM analysis.

Calcium and oxygen were present in the core and the rind, determined by Energy Dispersive X-ray Spectroscopy (EDX) after FIB milling (Supplementary Fig. S10a). The heterogeneous morphology of the precipitates, along with the EDX data and the autofluorescence might explain the presence of both vaterite and potentially calcite. Further crystallographic investigations are required in the future to better characterize the composition of the core and the rind.

### Bacteria as mineral scaffolds

**Figure 7:**
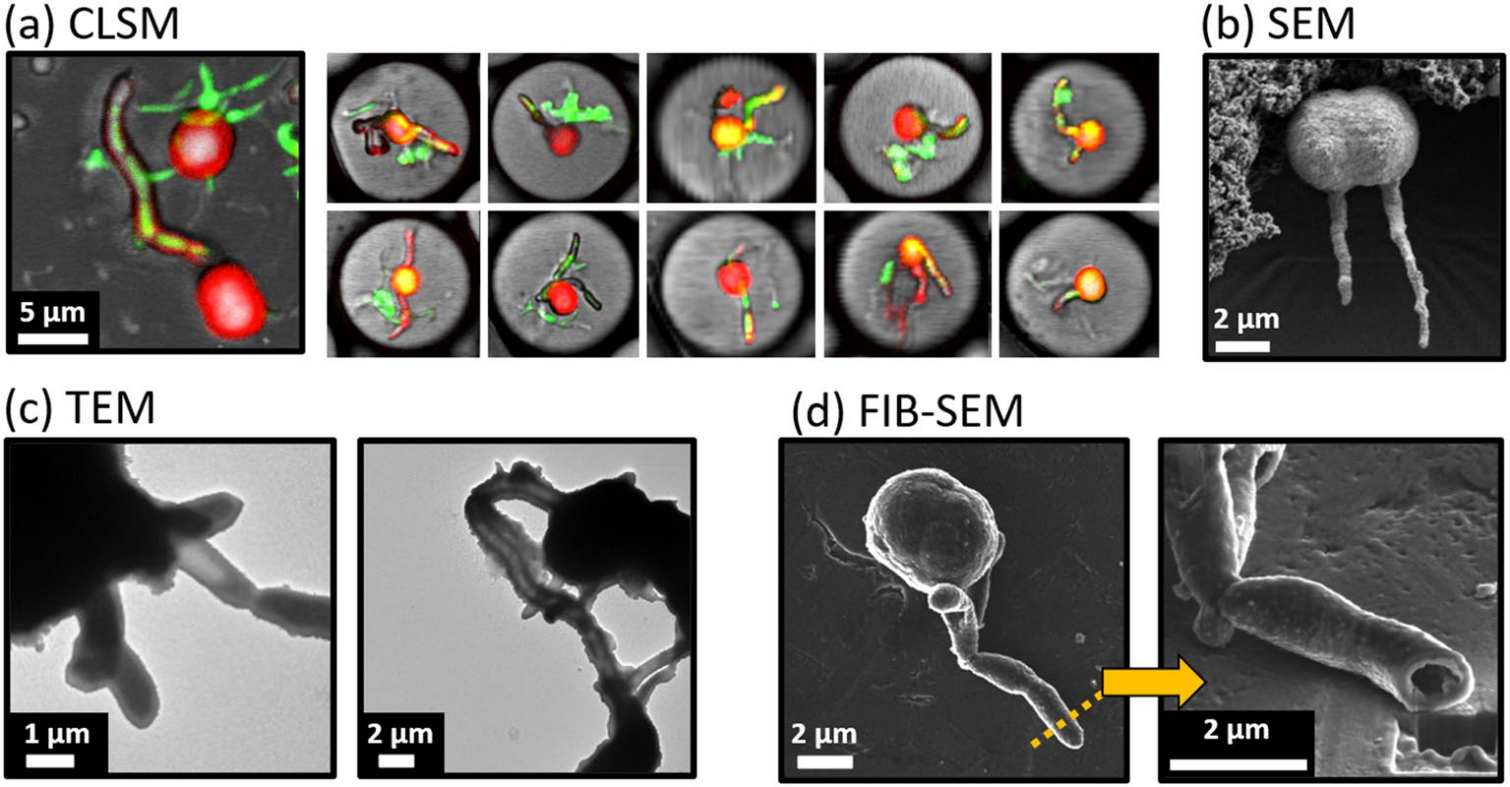
Images indicating cell encapsulation by precipitates. Cylindrical extensions observed using different imaging techniques. (**a**) CLSM images of drops with autofluorescent extensions (red) showing interior GFP signal (green) resembling the cell-chains displayed in Fig. 2. Individual drops shown are approximately 25 µm in diameter. (**b**) SEM image of a vaterite precipitate with two extensions. (**c**) TEM micrographs showing cell-shaped structures covered with precipitates. (**d**) FIB-SEM slicing of a cell-shaped extension indicating a hollow, possibly previously cell-occupied tube.

The mineralization and control experiments presented above show that bacterial ureolysis is a prerequisite for CaCO_3_ precipitation. Our microscopy data (Fig. 7) show that precipitates form on and around the bacteria themselves. Cylindrical sheaths of precipitate were observed closely surrounding bacterial cell-chains (Fig. 7). The sheaths were identified as CaCO_3_ based on their calcium and oxygen signals (NanoAuger EDX, Supplementary Fig. S10), and their autofluorescence when excited at 405 nm which is consistent with the autofluorescence of the precipitates observed throughout this study. The sheaths were approximately 1 µm in diameter and of variable length. In many instances, these precipitate sheaths showed a narrower, internal GFP fluorescence at 488 nm excitation (Fig. 7a), closely resembling the cell-chains seen in Fig. 2.

TEM images of the cell-shaped extensions suggested lower density structures resembling cells covered with precipitates (Fig. 7c). A cell-shaped extension was also cut and imaged using FIB-SEM (Fig. 7d); the interior appeared hollow, which is consistent with cell material either having degraded or collapsed due to the SEM vacuum conditions. The cut extension showed calcium and oxygen signals (Supplementary Fig. S10c). Overall, the microscopic investigations using SEM and TEM suggest that cells were encased in precipitates, and the fully hydrated CLSM images show that the encasement of cells in precipitates is not an artifact from sample preparation (drying, vacuum etc.), but rather that the precipitates are surrounding the cells in situ.

## Conclusions

Abiotically-driven calcium carbonate precipitation has been previously studied using drop-based microfluidics via mixing of calcium chloride and sodium carbonate solutions inside drops^22–24^. Biologically-driven precipitation, which is dependent on bacterial activity, has to our knowledge not been studied in drops before, and this work is the first to combine drop-based microfluidics with microscopy to visualize ureolysis-driven MICP starting from single cells.

Bacterial ureolysis was promoted in microscopic droplets containing single ureolytic cells. Calcium carbonate precipitates formed in the presence of the three prerequisites for MICP (ureolytic cells, dissolved urea and dissolved calcium). The different calcium ion concentrations tested in drops resulted in bacterial aggregates of different types, including clusters, chains, and clumps. Cell motility decreased over time in all conditions and higher calcium ion concentrations led to a decrease in initial cell motility. Precipitates in drops underwent morphological and mineralogical changes over time. Precipitates in drops that did not originally contain cells likely formed via transport of carbonate and/or hydroxyl ions between drops.

Our results indicate a combination of vaterite and potentially calcite as the final products. These precipitates had a Raman signal consistent with that of vaterite, but also exhibited an autofluorescence signal under CLSM, which indicated the presence of calcite. This was further inferred from FIB-SEM results obtained from the vaterite precipitates, which revealed a heterogeneous structure consisting of an interior core covered by a grainy rind, suggesting two different morphologies and thus possibly mineral phases.

The presence of organics likely aided in the formation and prolonged the stability of amorphous calcium carbonate and vaterite, both metastable CaCO_3_ polymorphs. The possible, partial phase transition to calcite, implied by the autofluorescence, could also be related to the presence of organics. For example, extracellular organic matrix-associated phosphoproteins have been shown to stabilize ACC^43^. Stable vaterite has been synthesized in the presence of root proteins of chick pea seeds^44^ and small proteins of different types of EPS can also influence the polymorphism of CaCO_3_ precipitates^45^. Most related to our produced precipitates is a study by Wang et al. in which vaterite microspheres with loops of calcite were generated in the presence of poly-sodium 4‐styrenesulfonate (PSS) and folic acid^46^. Organics can not only affect CaCO_3_ polymorph selection^47^ but extracellular organics such as glycoproteins and other macro molecules produced by microbes can also increase crystal aggregation^48^. Future studies will investigate the role of organics in phase transitions.

A microscopy-based approach involving thin-sectioning of the precipitates followed by spatially-resolved fluorescence, and TEM/SEM diffraction or Raman vibration-based characterizations of the thin sections did not yield conclusive results. Identifying the crystal phases of the core and the rind would allow for an in-depth understanding of mineral formation and transformation with the potential for controlling resulting material properties.

In summary, we observed precipitates surrounding bacterial cells through various microscopy methods, including CLSM, SEM, FIB-SEM and TEM. These observations support the hypothesis that bacterial cells can serve as mineral scaffolds and possibly nucleation points for precipitation and can become encapsulated in CaCO_3_ during MICP^10–13^. Correlative microscopy studies focused on characterizing the developing precipitates, specifically targeting temporal changes in morphology and mineralogy, will be performed in the future. The research presented here provides a platform for single-cell and single-crystal level studies as well as for the study of many other microbe-mineral interactions, such as other biomineralization processes, sequestration of heavy metals or radionuclides, bioleaching and mineral dissolution. Finally, the observed uniformity in size and shape of precipitate aggregates is highly promising for micro-manufacturing of small particulate minerals or nanoparticles with specific material properties.

## Supplementary information

Supplementary Information 1.

Supplementary Information 2.

Supplementary Video S1.

Supplementary Video S2.
