# Supplementary material for: Mineralogy of microbially induced calcium carbonate precipitates formed using single cell drop-based microfluidics
Source: Sci Rep. 2020 Oct 16;10:17535. doi: 10.1038/s41598-020-73870-y (PMC7568533; doi:10.1038/s41598-020-73870-y)
Supplement: Supplementary file 1 — Supplementary Information 1. [file 41598_2020_73870_MOESM1_ESM.pdf]

Supplementary Information for

Mineralogy of Microbially Induced Calcium Carbonate Precipitates Formed Using Single Cell Drop-Based Microfluidics

Neerja M. Zambare [1,2], Nada Y. Naser [1,2,3], Robin Gerlach [1,2], Connie B. Chang [1,2]**

[1]*Department of Chemical and Biological Engineering, Montana State University, Bozeman, MT 59717, USA*

[2]*Center for Biofilm Engineering, Montana State University, Bozeman, MT 59717, USA*

[3]*Department of Chemical Engineering, University of Washington, Seattle, WA 98195, USA*

Contact Information:
N.M.Z.: neerja.zambare@montana.edu
N.Y.N.: nynaser@uw.edu
R.G.*: robin_g@montana.edu
C.B.C.*: connie.chang@montana.edu
**Corresponding Authors*

Optimizing bacterial concentration to achieve 1 cell per drop

Escherichia coli MJK2 were grown overnight in growth medium and then diluted to an optical density of 0.4 (OD_{600}), measured using a BIOTEK Synergy HT spectrophotometer (200 µL samples in a 96-well plate, with the blank well OD of 0.044 as reference). The adjusted culture was serially diluted using growth medium (recipe in Materials and Methods) and plated on LB with 2% Urea agar as 10 ul drops.[1] Bacterial growth was assessed in terms of Colony Forming Units per milliliter (CFU/mL) using the number of colonies observed at 24 hours in each drop that was plated. The dilution of 10^{-8}, which resulted in colony counts of 1-15 per plated drop, was chosen for total bacterial concentration estimates. This protocol was performed on 25 separately grown overnight cultures with a resulting average bacterial concentration of 5.1 ± 1.3 x 10^8 CFU/mL. The desired concentration of cells for a single cell in drop was calculated to be 1.2 x 10^8 cells/mL for 25 µm diameter drops. Accordingly, the OD-adjusted culture was diluted 1:5 using growth medium prior to making drops.

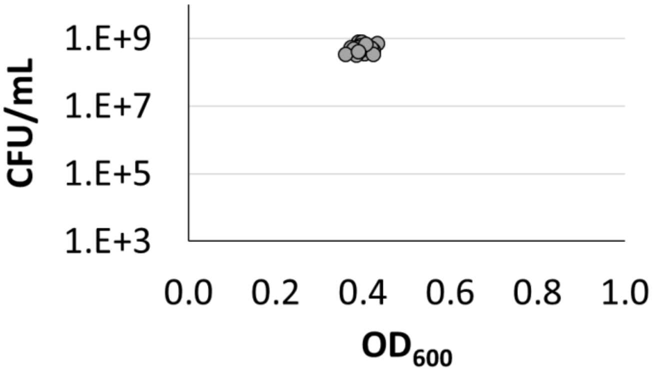

Figure S1: *Cell concentrations (CFU/mL) of 25 biological replicate cultures adjusted to an optical density of 0.4 (600 nm reading).*

	Urea-free	Ca^{2+}-free	Ca_{Low}	Ca_{Int}	Ca_{High}
Day 0	238	81	252	200	98
Day 1	200	212	219	199	221
Day 2	342	178	355	196	218
Day 3	447	200	402	122	221
Day 4	390	189	402	187	177

Numbers of drops analyzed for growth curves (Figure 3)

Table S1: *analyzed on condition for*

Number of drops each day for each growth curves in Figure 3.

	Urea-free	Ca^{2+}-free	Ca_{Low}	Ca_{Int}	Ca_{High}
Day 0	157	710	261	220	52
Day 1	228	1326	1079	162	46
Day 2	99	451	460	69	14

Number of motility tracks analyzed (Figure 3)

Table S2: *Number of motility tracks detected in drops containing cells on each day for each condition. The averages and standard deviations for velocities of tracks for each day and condition are plotted as swimming speeds shown in Figure 3.*

Drop count differentiation for the two precipitate morphologies

Two precipitate morphologies were observed in the drops in these experiments. The number of drops counted at the 25 µm diameter detection setting for the reflection signal provided the total number of drops containing precipitate (either morphology). Drop tally measurements with a diameter detection setting of 5 µm selected for the drops containing the smaller vaterite precipitates. The difference in the total number of precipitate-containing drops and the number of vaterite-containing drops resulted in the number of ACC-containing drops.

Correlation between OD_{600} and GFP for *E. coli* MJK2

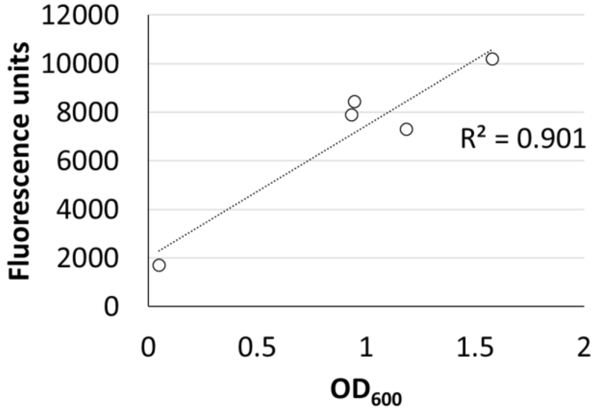

Figure S2: *Correlation between OD_{600} and GFP-based fluorescence for E. coli MJK2 growing in Ca-free media.*

Fluorescence measurements in bulk studies

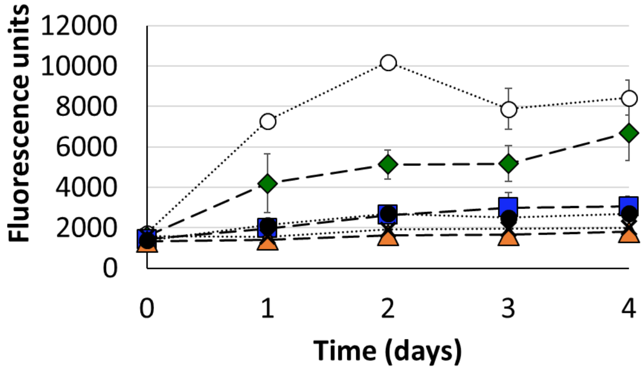

Figure S3: Cell growth in 10 mL bulk experiments as a function of time, in terms of GFP intensity measurements on a plate reader for Ca^{2+}-free (○), urea-free (●), bacteria-free (×), Ca_{Low} (◆), Ca_{Int} (■) and Ca_{High} (▲).

Difference in fluorescence trend for Ca_{Int}

In the Ca_{Int} drops, the GFP signal initially increased but then decreased drastically after day 1 and continued to decrease over time (Fig. 3.a). This decrease in fluorescence after day 1 was not observed in bulk experiments but was confirmed by measuring GFP intensity in drops from four biological replicates of the Ca_{Int} condition (Supplementary Fig. S4). A potential reason for the discrepancy between GFP signals from drops and bulk experiments for the Ca_{Int} condition could be due to the difference in optics between the CLSM and the fluorescent plate reader used for the drop and bulk experiments, respectively. Precipitates settle to the bottom in both, drop and bulk experiments, as observed in Supplementary Video S2 for drops. In drop experiments, the settled precipitates can attenuate the fluorescence signal from bacterial cells as the excitation laser source and detector in the inverted CLSM are both located below the specimen. Bulk experiments were performed in centrifuge tubes where the precipitates attach to the side of the centrifuge tubes and settle to the bottom. Samples were then taken from the fluid phase for fluorescence quantification using a 96-well plate reader. Suspended precipitates settle to the bottom of the well plate, and since the excitation source and detector are located above the well plate, the precipitates will not or only slightly affect the fluorescence measurements using the plate reader. Since the Ca_{Int} drops showed the greatest number of precipitates

that had settled to the bottom of the drops on day 2 and onwards (discussed in section '*Precipitation in drops*'), the interference of the precipitates with the fluorescence measurements would indeed be greater in this condition compared to the other conditions and could explain the decrease in GFP signal observed only in the Ca_{Int} drops.

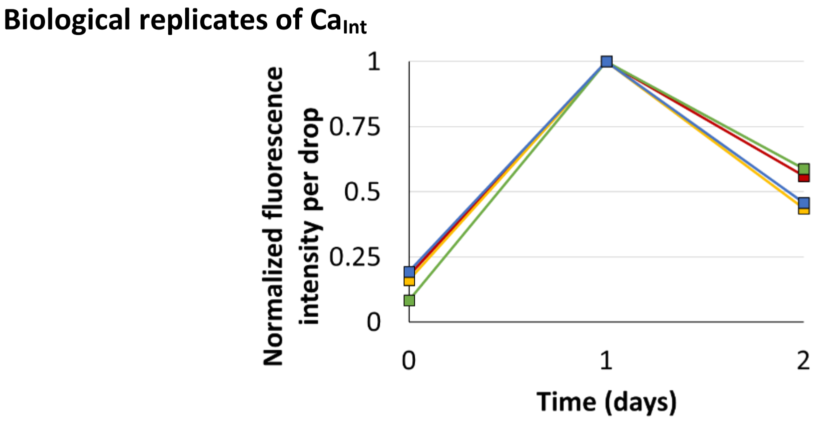

Figure S4: *Fluorescence intensity per drop normalized to the respective maximum value for each biological replicate of Ca_{Int} over time. The maximum fluorescence signal is measured on Day 1 for all replicates, followed by a decrease in fluorescence signal. The colors correspond to four replicates.*

Precipitate morphologies in the drops

Images from Ca_{Int} drops show precipitates in drops over time (Figure S5). The top row shows brightfield, GFP and autofluorescence signal overlays (channels (i), (ii) and (iii) described in Materials and Methods). The bottom row shows the corresponding reflection signals (channel (iv)). The first precipitates detected had a reflection signal but no corresponding autofluorescence. The autofluorescent precipitates formed in drops with cells but were also seen in drops without GFP signal.

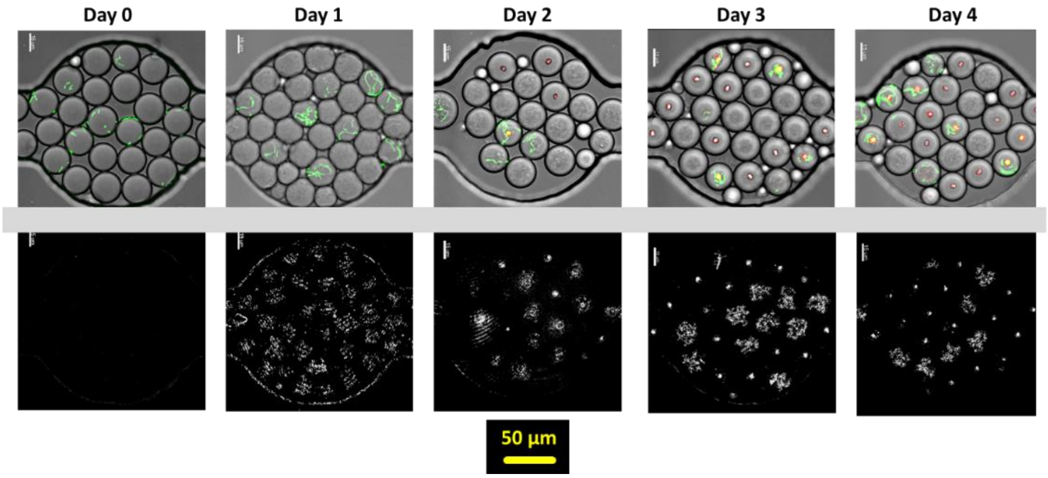

Figure S5: *Drops showing cells (GFP, green) or precipitates (autofluorescence, red) for the Ca_{Int} condition. The bottom row shows the corresponding reflection signals from the drops in the top row. Two visually distinct morphologies of precipitates are observed via reflection imaging.*

Bulk Ureolysis

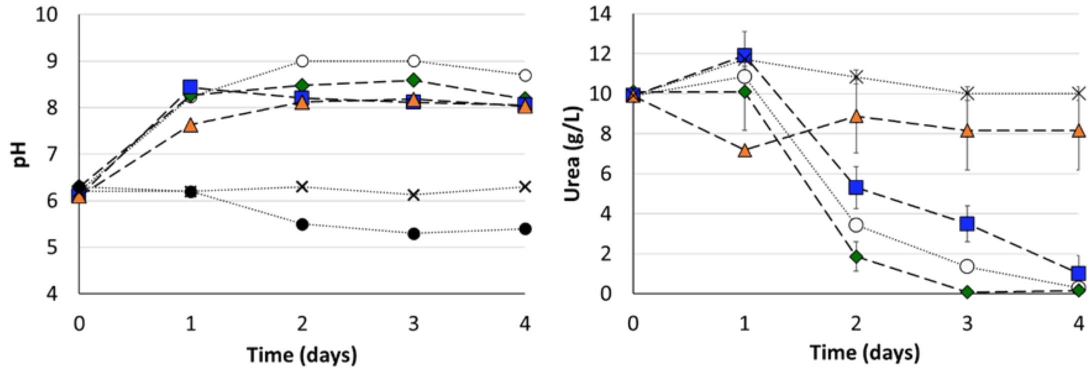

Figure S6: (a) *pH measurements and* ***(b)*** *urea concentrations in tube reactors over time for Ca^{2+}-free (○), urea-free (●), bacteria-free (×), Ca_{Low} (◆), Ca_{Int} (■) and Ca_{High} (▲).*

An increase in pH is an indicator for ureolysis (Equations 1 and 2). Figures S6.a and S6.b show evidence of ureolysis in Ca^{2+}-free, Ca_{Low}, Ca_{Int}, and Ca_{High}. Of these experiments, Ca^{2+}-free, Ca_{Low}, and Ca_{Int} showed near-complete removal of urea while at the high Ca concentration not all urea was removed. Ureolysis was slower in Ca_{High} likely due to lower cell growth observed at higher calcium concentrations (*cf*. Figure S3). As expected, the bacteria-free and urea-free controls did not hydrolyze urea and did not show an increase in pH.

Formation of precipitates in drops without bacteria

In experiments with bacteria, precipitates were also observed in drops which did not contain bacteria (Figures 4.a and S5). Hence, it was hypothesized that diffusion of carbonate and/or hydroxyl-ions occurred from drops with actively urea-hydrolyzing cells to bacteria-free drops. To test this hypothesis, control drops containing only fluorescent microbeads ((Excitation at 488 nm/ Emission collected above 600 nm, ThermoFisher Scientific, MA, USA) and dissolved calcium were generated. These control drops did not contain bacteria or urea, two of the three prerequisites for MICP, which are actively urea-hydrolyzing cells, urea and calcium. These control drops were mixed with Ca_{Int} drops containing ureolytic cells (50:50 mixture by volume) and incubated. After three days, precipitates were observed in all drops regardless of whether they initially contained ureolytic cells (Figure S7). Since no precipitates had formed in completely bacteria-free control experiments (Figure 2 in manuscript), this experiment demonstrates that diffusion of ureolysis-generated products (e.g. carbonate and/or hydroxyl-ions) between drops is possible and therefore, precipitation of calcium carbonate occurs in drops that did not originally contain actively urea-hydrolyzing cells.

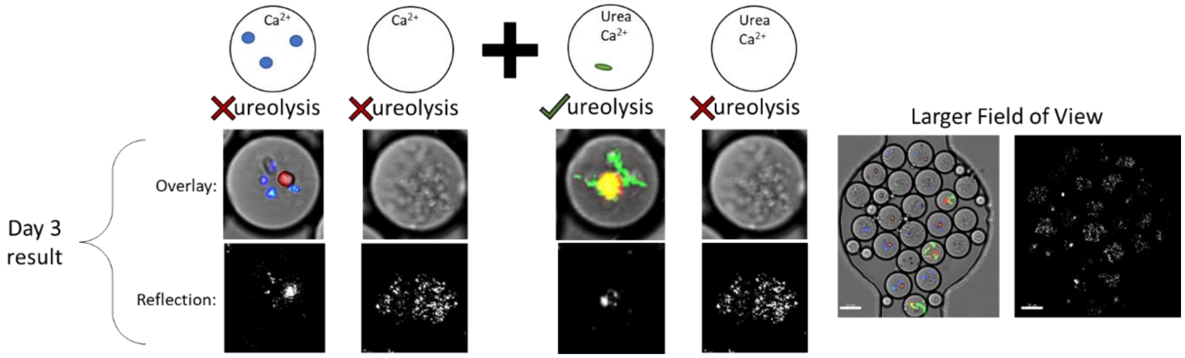

Figure S7: *Experimental schematic of drops containing fluorescent microbeads (blue) and dissolved Ca mixed in 50:50 proportion (by volume) with Ca_{Int} drops. The corresponding results of each drop scenario in the schematic after three days is provided below. A larger field of view is provided on the right. Similar to generation of drops with bacteria where only 1 in 10 drops generated contain a single cell, not all control drops contain microbeads. Note that for drops with dissolved calcium and no beads, and for drops with dissolved urea and calcium and no bacteria, the result shown is the same. This is because it is impossible to differentiate between these drops using the microscopy techniques employed as they do not have a detectable indicator such as microbeads or cells. Precipitates (detected by reflection signal) appeared in all drops, even those that did not contain bacteria.*

X-Ray Diffraction data for vaterite standard in Figure 5

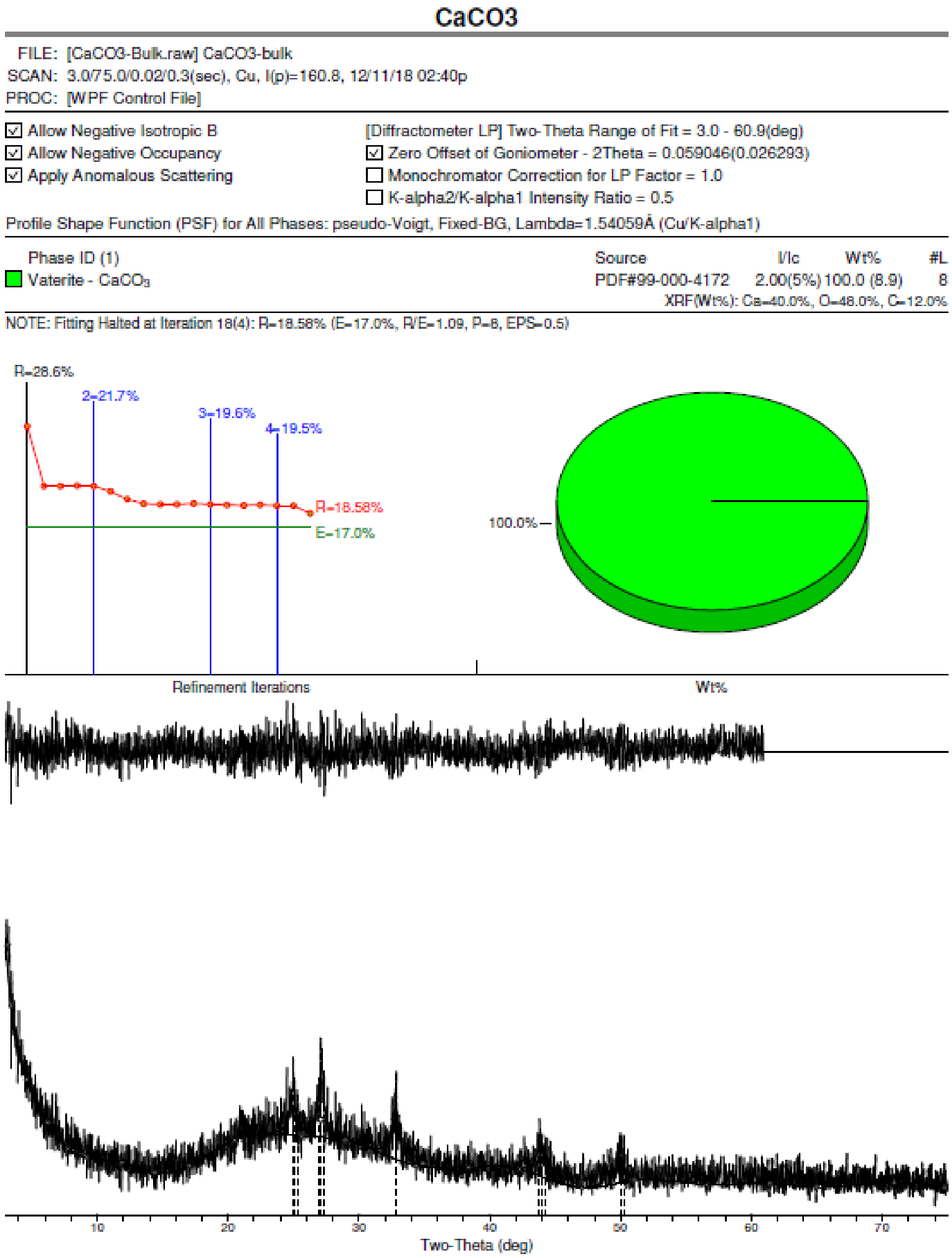

Figure S8: *XRD characterization of a lab-generated precipitate sample from a flask containing MJK2 and mineralization media using a Scintag X1 Diffraction System. The precipitates were characterized as vaterite by XRD.*

Fluorescence emission scans

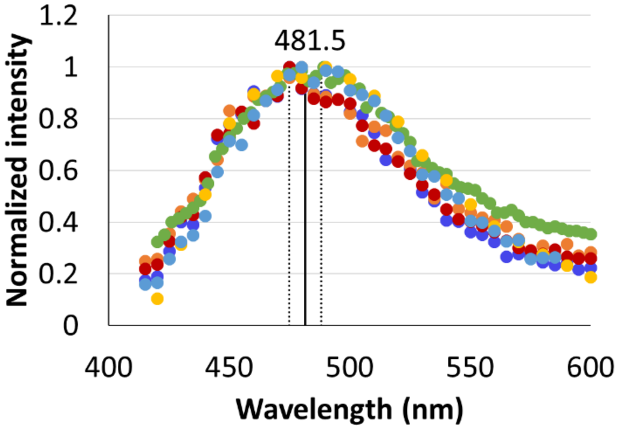

Figure S9: *Fluorescence emission scans collected from six randomly chosen vaterite precipitates from Ca_{Int} drops (405 nm excitation).*

Figure S9 shows fluorescence emission scans (405 nm excitation) from six randomly chosen vaterite precipitates from Ca_{Int} drops. The y-axis is normalized so a value of 1 signifies the maximum fluorescence intensity, which was observed at an average emission wavelength of 481.5 ± 6.6 nm (shown by vertical lines on plot).

Presence of Ca in precipitates and on the extensions

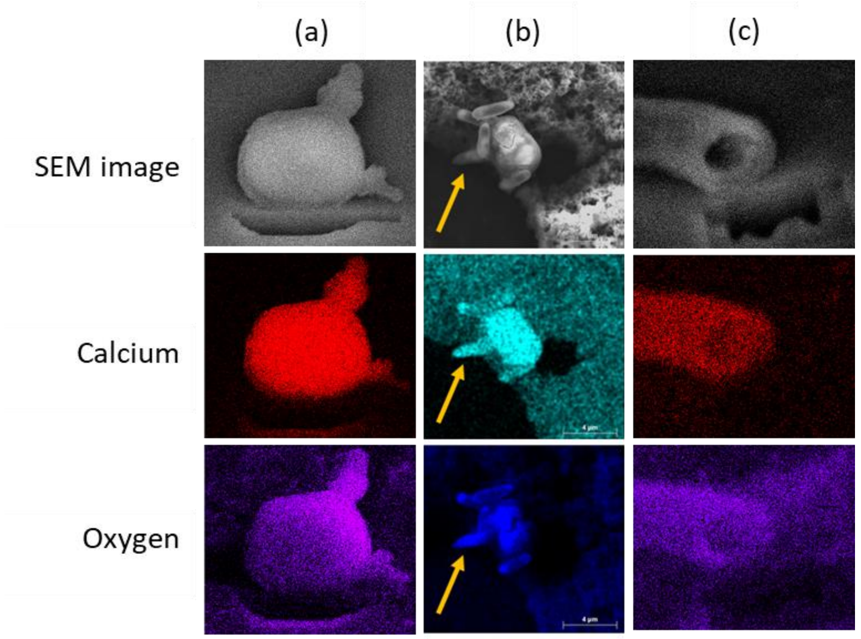

Figure S10: *EDX maps for calcium and oxygen taken from **(a)** the interior of a precipitate during milling by FIB-SEM, **(b)** an area showing one vaterite precipitate and some cell-type structures and **(c)** a milled cell-like extension.*

Supplemental Videos

Video_S1.mp4

Video S1: *Movie showing all channels overlaid (channels (i), (ii), (iii) and (iv) listed in methods). The movie shows, over 2 minutes of acquisition time, the close spatial association of cells and precipitates.*

Video_S2.avi

Video S2: *Frames moving sequentially from the top of the drops (PDMS side) to the bottom (glass cover slip side). The vaterite precipitates appear predominantly at the bottom of the drops. Frames are from a z-stack with z-step resolution of 0.8 µm.*

REFERENCES

1 Herigstad, B., Hamilton, M. & Heersink, J. How to optimize the drop plate method for enumerating bacteria. *Journal of Microbiological Methods* **44**, 121-129 (2001).
