# Supplementary material for: Mineralogy of microbially induced calcium carbonate precipitates formed using single cell drop-based microfluidics
Source: Sci Rep. 2020 Oct 16;10:17535. doi: 10.1038/s41598-020-73870-y (PMC7568533; doi:10.1038/s41598-020-73870-y)
Supplement: Supplementary file 2 — Supplementary Information 2. [file 41598_2020_73870_MOESM2_ESM.docx]

**Supplementary Information**

Summary of optimizing bacterial culture conditions to achieve 1 cell per drop (Fig. S1), numbers of drops analyzed for growth and motility measurements (Tables S1 and S2), Drop count differentiation method for the two precipitate morphologies, OD_600_ and GFP correlation (Fig. S2), fluorescence growth curves for bulk experiments (Fig. S3) and discussion of the difference in fluorescence trend for Ca_Int_, biological replicates of Ca_Int_ drops (Fig. S4), precipitate morphologies in drops (Fig. S5), ureolysis in bulk experiments (Fig. S6), and formation of precipitates in drops without bacteria (Fig. S7). Also provided are XRD data for the vaterite standard (Fig. S8), fluorescence emission spectra for precipitates (Fig. S9), elemental maps of calcium and oxygen in precipitates and on precipitate extensions (Fig. S10), and videos of cells and precipitates in drops (Videos S1 and S2).
